# Proteomics progresses in microbial physiology and clinical antimicrobial therapy

**DOI:** 10.1007/s10096-016-2816-4

**Published:** 2016-11-04

**Authors:** B. Chen, D. Zhang, X. Wang, W. Ma, S. Deng, P. Zhang, H. Zhu, N. Xu, S. Liang

**Affiliations:** 1 State Key Laboratory of Biotherapy and Cancer Center, West China Hospital, Sichuan University, and National Collaborative Innovation Center for Biotherapy, No. 17, 3rd Section of People’s South Road, Chengdu, 610041 People’s Republic of China; 2 Department of Urinary Surgery, West China Hospital, West China Medical School, Sichuan University, Chengdu, 610041 People’s Republic of China; 3 Laboratory of Cell and Molecular Biology & State Key Laboratory of Molecular Oncology, Cancer Institute & Cancer Hospital, Chinese Academy of Medical Sciences, Beijing, 100034 People’s Republic of China

## Abstract

Clinical microbial identification plays an important role in optimizing the management of infectious diseases and provides diagnostic and therapeutic support for clinical management. Microbial proteomic research is aimed at identifying proteins associated with microbial activity, which has facilitated the discovery of microbial physiology changes and host–pathogen interactions during bacterial infection and antimicrobial therapy. Here, we summarize proteomic-driven progresses of host–microbial pathogen interactions at multiple levels, mass spectrometry-based microbial proteome identification for clinical diagnosis, and antimicrobial therapy. Proteomic technique progresses pave new ways towards effective prevention and drug discovery for microbial-induced infectious diseases.

## Introduction

Infection is a leading cause of death around the world, which has especially become a growing threat for developing countries. More than 50% of emerging infectious diseases are caused by bacteria or rickettsia, including a large number of drug-resistant microbes [1]. Clinical microbial identification includes the confirmation of bacterial, viral, fungal, and parasitic agents that cause human disease. The precise identification for microbial pathogen provides diagnostic and therapeutic support for the clinical management of patients, surveys local and global epidemiology, as well as helps to prevent the infectious diseases transmission [2]. With the emergence of resistant strains and the release of large amounts of antimicrobials, anti-infection drugs are being severely tested [3]. Microbial resistance to antibiotics is on the rise and, yet, few new antibiotics active against multiresistant bacteria are being explored [4, 5]. New antibiotic agents against microbial infections need to be developed to tide over this crisis [6]. Microbial physiology usually focuses on biofilms and cell-wall biosynthesis, protein biosynthesis, DNA and RNA replication, folate metabolism, cell-surface decoration, and isoprenoid biosynthesis, from which researchers discern microbial molecular behaviors to explore drug targets for antimicrobial therapy [7, 8].

Proteomic studies are currently being greatly engaged in the microbial field [8, 9]. Proteomics could yield not only the qualitative information on proteins, including the identification, distribution, posttranslational modifications, interactions, structure, and function, but also quantitative information, like abundance, distribution within different localizations, and temporal changes in abundance due to synthesis and degradation or both [10, 11]. Microbial proteomic research is aimed at identifying proteins associated with microbial activity. By using gel-free and gel-based methods in combination with liquid chromatography (LC) and mass spectrometry (MS)-based techniques, it has become a formidable tool for deciphering microbial proteins [12]. By identifying the resistance genes towards antibiotics using the comparative proteome analysis for model strains and resistant mutants, microbial proteomic investigation would be helpful not only in instructing the clinical application, but also in the screening of potential bioactive compounds and new antimicrobial drugs [7, 13]. The proteomic analysis for biofilm provides a new idea of an antibiotic cocktail therapy strategy for infection [14]. Current MS-based proteomics technologies have advanced to the point where they are amenable to any biological system [15]. For example, protein isolation approaches, including affinity purification and tandem affinity purification, combined with MS are powerful tools to decipher new protein–protein interactions [16]. The renewed interest in microbial proteome profiling is to reveal the dynamics of microbiome [17]. So, here we summarize and present an overview of proteomic progress towards host–microbial pathogen interactions at different levels, and MS-based microbial identification for clinical diagnosis and antimicrobial therapy as follows.

## New insights into host–microbial pathogen interactions by proteomic tools

**Fig. 1:**
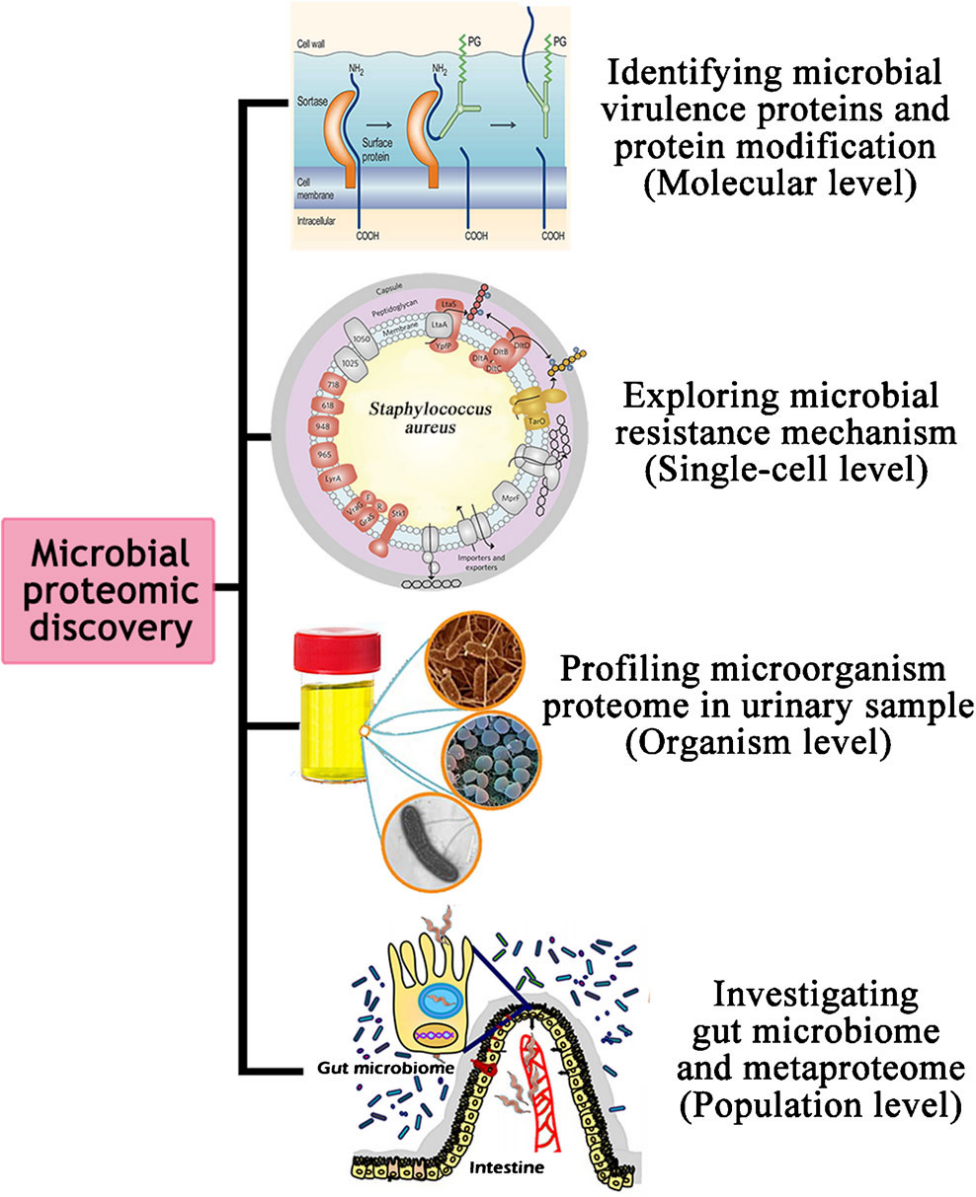
Host–microbial pathogen interactions from proteomics dissection

Interactions between the host and microbial pathogen are crucial for infections caused by microorganisms. Knowledge of these interactions, such as how microbial pathogens display their virulence to the host and develop their resistance, is, therefore, essential in order to better understand and develop strategies to fight infections. The new insights into host–microbial pathogen interactions by proteomic tools will be discussed at different levels, including molecular, single-cell, organism, and population levels (Fig 1).

### Identifying microbial virulence proteins and protein modifications

There are complex and dynamic interactions between pathogens and host immune defense mechanisms during the course of invasive infection, which could determine the fate of the host at the outset of the infection process [18]. Microbial pathogens subvert various molecules for their adhesion and invasion to host cells, infection of neighbor cells, dissemination into host systemic circulation, and evasion of host defense mechanisms. Proteomic profiling of the outer and inner membrane proteins and secreted proteins, such as siderophores, provided new insights into host–pathogen interactions [19]. Virulence proteins, like proteoglycans [20], mediate protein–pathogen interactions, to affect the onset, progression, and outcome of infection [21, 22]. Accumulating evidences indicate that microbial virulence contributes to host response and the outcome of severe infections [23]. For example, *Staphylococcus aureus*, a Gram-positive commensal bacterium, which has an extensive arsenal of virulence factors, is a major threat to modern healthcare systems. Moreover, some pathogens acquire the capacity to communicate with each other and sense the host’s vulnerabilities [24].

Moreover, protein modifications, including glycosylation, phosphorylation, and acetylation, seem to confer virulence, which can be rapidly identified by MS [25–27]. One popular human pathogen, *Mycobacterium tuberculosis*, has been investigated as a model microorganism using proteomic methods for over a decade; especially, hundreds of putative virulence determinants and posttranslational modifications have been identified [28]. Recently, PhoP, a highly conserved virulence-regulating protein in bacteria, has been confirmed to acetylate at the lysine residue 201 in *Salmonella typhimurium*, and it is deacetylated by deacetylase CobB enzymatically. Also, its acetylation causes significantly attenuated intestinal inflammation and systemic infection in the mouse model [29]. These findings on bacterial protein modifications ultimately lead to better management of the related disease.

It is noticed that a new branch of host–pathogen interactions at the atomic level is attempted to explore more microcosmic changes. The pioneer, Salgado, tried to determine the assembly and structure of the mature S-layer in *Clostridium difficile* to discover host–pathogen interactions at the atomic level [30]. The atomic level insight to microbial physiology will greatly enlarge our understanding for infectious disease.

### Exploring microbial resistance genes at the single-cell level

A single cell represents the basic unit of a living organism. To avoid heterogeneity in the function and fate of cell populations, it is vital to measure quantity and dynamic processes in single cells [31, 32]. Ultimately, the cellular plasticity depends on changes in protein expression levels and proteomic methods allowed to measure many proteins in parallel [33]. The emergence of resistant strains and the release of large amounts of antimicrobials are serious problems. To fill the multiple gaps that remain in understanding microbial resistance, proteomic tools have also been used to study microbial physiology in response to antibiotic stress [34]. Identification of the resistance genes against antibiotics by comparative proteome analysis of model strains and resistant mutants would be helpful not only in instructing the clinical application, but also in the evaluation of new drugs [7]. Moreover, proteomics technologies have also successfully unraveled the drug resistance mechanisms of microbial biofilms and possibly contributed to the new knowledge for future development in the field [13]. Our recent study identified the changed bacterial proteins of host strain *S. aureus* in response to daptomycin antibiotic treatment, which disrupts bacterial physiology at multiple levels [35]. And the findings help to develop novel daptomycin derivatives against the upcoming antibiotic-resistant bacterial infection.

Microbial proteomics also offer new approaches to develop potential bioactive compounds. The specific enzymes and proteins, non-ribosomal peptide synthetases and polyketide synthases, which are involved in the synthesis of natural products, are rapidly identified by proteomic analysis [31]. Proteomic methodologies contribute towards determining antimicrobial resistance genes; novel antibiotics designed targeting resistance genes will bring an important breakthrough of antibiotic development [36]. Especially, single-cell proteomics can identify proteins and measure protein concentrations directly in a single cell [37], which is a more powerful tool to pursue microbial resistance dynamics from a complex sample.

### Profiling microbial proteome at the organism level

As an important non-invasive body fluid source for diagnostic and prognostic biomarkers of human diseases, urine may contain whole human cells shed into the urine from anatomically proximal tissues and organs (e.g., kidney, prostate, bladder, urothelium, and genitals) [38, 39]. The cells derived from such tissues can viruses and microbial organisms which caused the urogenital tract infection. Identification of the function, abundance, and tissue of origin of such proteins could help to understand the host–pathogen interaction process, including the cause of urinary tract infection, and the human immune response to the infection-associated pathogen(s) [40]. For example, a study has reviewed the proteomic results of *Shigella dysenteriae*, *Shigella flexneri*, enterohemorrhagic *Escherichia coli*, and uropathogenic *E. coli* [41]. It showed that the nutrient availability and oxygen had dynamic adaptations to changes, including the increased anaerobic respiration and mixed acid fermentation in vivo. And the host model investigated mainly determined the utilization of carbon and nitrogen resources by the bacteria.

### Investigating the community physiology and pathogenicity at the population level

Recent studies have shown that the local contact or social population structure of the host may cause large shifts in virulence in pathogen populations as a result of a bistability in evolutionary dynamics [42]. Mixtures of thousands of different phylotypes interact with each other and with their environment [40, 43].

Advances in host–pathogen interactions by proteomic tools at the population level were well illustrated by the development of metaproteomics. The emerging field of metaproteomics aims at analyzing the proteome profiles of mixed microbial communities, from which community physiology and pathogenicity are learnt [44, 45]. Metaproteomics analyzes the abundance and activity of enzymes during nutrient cycling to their phylogenetic origin at the protein level [46, 47]. Metaproteomics opens a door to capture the natural products from uncultivated microorganisms into model production host strains, recognizing the metabolic spectrum of microbes that are not fully expressed in laboratory culture and which could not be sampled by classical means [40, 46]. Multispecies bacterial biofilms of the catheter were dissected by a metaproteomics approach [48], which unraveled the bacterial community structure and function of the related biofilm, elucidating the interplay between bacterial virulence and the human immune system within the urine.

Metaproteomics technology has made a direct impact on our understanding of microbial diversity, ecology, and secondary metabolism, which would provide an efficient guide to the access of numerous non-culturable microorganisms for their associated prosperity for potential applications in clinical biomarkers screening and natural product antibiotic discovery. One group has adopted shotgun metaproteomic approaches combined with metagenomics to identify potential functional signatures of Crohn’s disease in stool samples [49]. Their study revealed the genes, proteins, and pathways that primarily differentiated subjects with Crohn’s disease in the ileum from the healthy patients and underscored the link between the gut microbiota and functional alterations in the pathophysiology of Crohn’s disease, aiding the identification of novel diagnostic targets and disease-specific biomarkers. Similarly, metabolomics has also been applied to discover the biomarkers of hepatocellular carcinoma [50] and catheter-associated urinary tract infections. For example, proteins related to pathogenicity and resistance/survival, beta-lactamase and TetR, are detected by metaproteome analysis, which may assume special relevance in terms of pathogenesis and resistance to host defenses and treatment [45].

More and more investigations into pathogens focus on gut microbiome and human health [51, 52]. Indeed, the microbiome is intrinsically complex, with many important functions. Mammalian gut microbiota is considered to be a novel type of “organ” [53, 54]. A more recent article has shown how fundamentally important the intestinal bacteria are to the rest of our mental and physical health, affecting almost everything from our appetite to our state of mind [55]. Study in microbial populations opens up a new research area in which researchers can get more relevant details. With this trend, the White House Office of Science and Technology Policy, in collaboration with federal agencies and private-sector stakeholders, announced the National Microbiome Initiative (NMI) on May 13, 2016. The NMI will launch with a combined federal agency investment of more than $121 million in fiscal year 2016 and 2017 funding for cross-ecosystem microbiome studies, aiming to foster the integrated study of microbiomes across different areas, such as healthcare, food production, and environmental restoration.

However, there are still some critical obstacles that need to be addressed. Proteomics identified peptides by matching MS/MS spectra against theoretical spectra of all candidate peptides represented in a reference protein sequence database [56]. The subsequent inference of the protein identity and protein quantification using the sequences and abundances of the identified peptides is based on a reference protein sequence database, such as Ensembl, RefSeq, and UniProtKB [57]. Nevertheless, these databases may not contain all the peptides and many peptides may not present in any reference database. Besides, peptides may contain mutations and may represent novel protein coding loci or alternative splice forms [58]. Alternatively, the proteogenomics approach was introduced in 2004 [59], using proteomic data derived from MS to improve and refine genome annotation. A number of automated softwares for proteogenomic analyses have been developed for the integration of MS-based proteomic data into genome databases [60–63]. For example, a one-stop open-source software termed GAPP, which applies the target-decoy search strategy to calculate the false discovery rate (FDR) for all employed algorithms’ results, provides a large-scale posttranslational modifications analysis on a proteome-wide level against prokaryotes [64].

## MS-based proteomics progresses on microbial identification and antimicrobial therapy discovery

### MS-based methods for rapid identification of clinical microbials

**Fig. 2:**
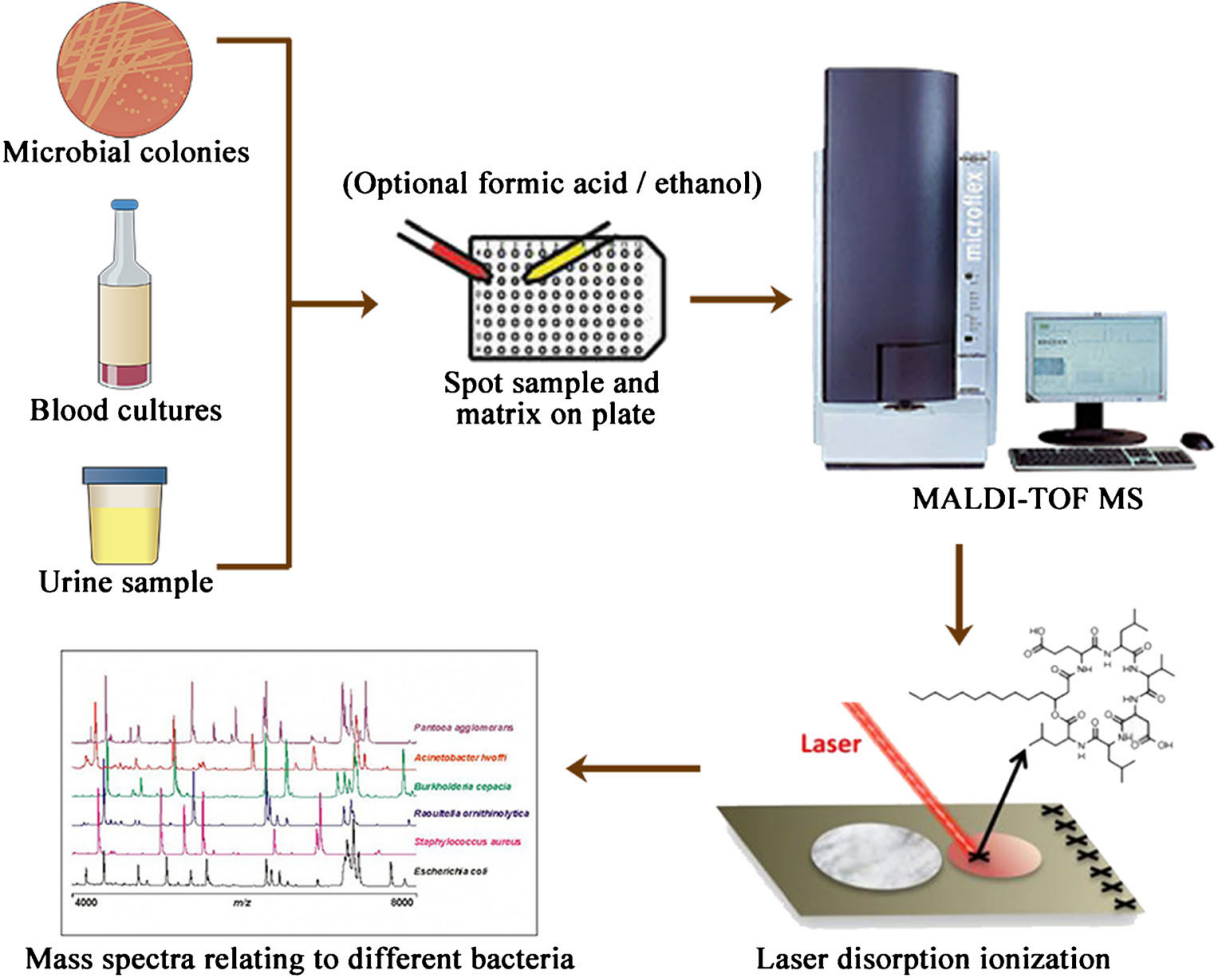
A simplified illustration for the general analysis of clinical microbials by matrix-assisted laser desorption/ionization time-of-flight mass spectrometry (MALDI-TOF MS). MALDI-TOF MS allows the identification of microbial pathogens cultured on agar, in blood culture bottles, or directly from urine samples. After being spotted on the plate, the sample is covered with a matrix and then desorbed and ionized by a laser to generate a specific fingerprint. To improve the spectral generation, formic acid and ethanol-based methods are optional. A fingerprint pattern is searched against a microbial standard library for the most matching spectra

Traditional identification of bacterial isolates has long relied on a combination of biochemical properties such as oxygen requirement, Gram staining, carbohydrate metabolism, and the presence of specific enzymes [3]. Nowadays, MS-based proteomic approaches have been used regularly in routine clinical diagnostic procedures, including the comprehensive characterization, classification, and identification of microorganisms [65–67]. Matrix-assisted laser desorption/ionization time-of-flight MS (MALDI-TOF MS) has been broadly adopted by many clinical microbiology laboratories over the past decade [68, 69]. The general schematic for the analysis of microbiological isolates and clinical material is illustrated in Fig. 2. MALDI-TOF MS is a very reproducible and reliable tool for microbial identification and can identify bacterial isolates in a few minutes and with low costs, with high efficiency from both a diagnostic and a cost-per-analysis point of view [70, 71]. Currently, a commercial MALDI-TOF MS system (VITEK® MS) has been approved by the U.S. Food and Drug Administration after extensive and successful clinical trials predominantly [72].

Two-dimensional gel electrophoresis (2-DE), which is no longer the exclusive separation tool used in the field of proteomics but still offers the highest resolution in protein separation, was typically combined with MALDI-TOF MS for microbial protein identification by peptide mass fingerprinting [73–75]. Moreover, in combination with isolation techniques, MALDI-TOF MS can be used to identify bacteria directly from blood culture bottles and urine samples [72, 76–78]. The MS identification of *Candida* species directly from blood culture bottles within 30 min was concordant with the conventional culture-based method for 95.9% of *C. albicans* and 86.5% of *Candida* non-*albicans* [72]. Moreover, mass cytometry, namely flow cytometry coupled with mass spectrometry, has been applied to rapidly process urine samples [77, 78].

**Fig. 3:**
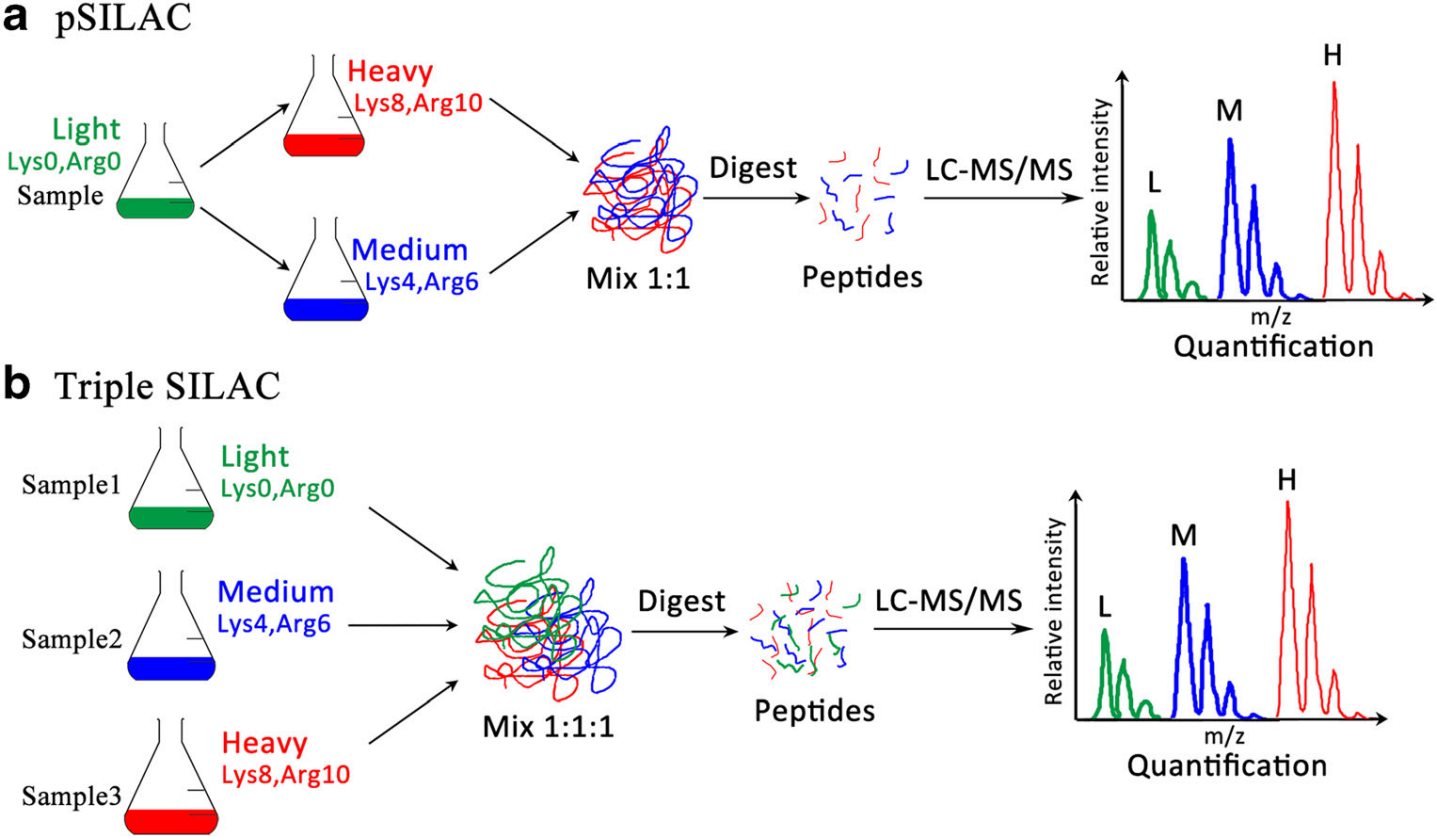
Stable isotope labeling with amino acids in cell culture (SILAC)-derived metabolic techniques. **a** pSILAC (pulsed SILAC) incorporated the stable isotope into proteins by adding “heavy” amino acids into the growth media. Cells are experimentally manipulated while growing in “light” (Lys0, Arg0) SILAC medium. Subsequently, the treated and control samples are transferred to distinctly labeled SILAC media, “heavy” (Lys8, Arg10) and “medium” (Lys4, Arg6). After one or a few doublings, samples are harvested and combined at the ratio 1:1. Proteins present before treatment will show up as a “light” peak (L) in the mass spectrograph and can be ignored. The effect of the treatment on protein production rates can be calculated as the ratio of signal at the “medium-heavy” (*M*) and “heavy” (*H*) peaks. **b** In triple SILAC, three samples can be analyzed at the same time, labeled with “light” (Lys0, Arg0), “medium” (Lys4, Arg6), and “heavy” (Lys8, Arg10) SILAC medium. Proteins were then combined and analyzed together by liquid chromatography tandem mass spectrometry (LC-MS/MS). In the MS spectra, each peptide appears as a triplet with distinct mass differences. The ratios between the samples are calculated directly by comparing the differences in the intensities of the peaks. “Lys0, Arg0”: unlabeled lysine and arginine; “Lys4, Arg6”: ^2^H_4_-lysine and ^13^C_6_-arginine; “Lys8, Arg10”: ^13^C_6_^15^N_2_-L-lysine and ^13^C_6_^15^N_4_-L-arginine

Stable isotope labeling with amino acids in cell culture (SILAC), a widely used in vivo metabolic labeling method, incorporates a stable isotope into the proteins in vivo by adding an isotope like ^13^C, ^15^N, or ^18^O as salts or amino acids to the growth media [79]. Of course, new SILAC-based approaches have been updated to improve identification efficiency. Through the *E. coli* cell-free protein expression system, named PURE (protein synthesis using recombinant elements) [80], the preparation of stable isotope-labeling reference peptides is performed in a 96-well plate within a short period. This SILAC labeling system, based on the reconstituted *E. coli* translation machinery, offers a general and rapid cell-free SILAC approach, which is also applicable for microbial MS identification. With extensive modification of the SILAC method, a pulsed SILAC (pSILAC) has been developed to monitor modest changes of proteins during de novo protein synthesis by metabolic pulse labeling of cells using two different heavy isotopic forms of arginine and lysine [81]. Meanwhile, the triple SILAC method, accomplished by SILAC in a triple labeling format (Fig 3), allows to study proteins derived from three samples or the time dimension of the proteome [82, 83]. These methods widely broaden the scope of SILAC-based proteomics.

### Quantitative proteomics techniques applied for monitoring antimicrobial therapy

Advanced methods of quantitative proteomics are capable of quantifying proteins and peptides from microbial strains with high resolution, which is available for dynamically monitoring microbial changes and drug efficiency. The selected reaction monitoring (SRM) is a greatly effective method, in which an ion of a particular mass is selected in the first stage of a tandem MS and an ion product of a fragmentation reaction of the precursor ion is selected in the second MS stage for detection [84]. Multiple reaction monitoring (MRM) is the application of selected reaction monitoring to multiple product ions from one or more precursor ions. MRM/SRM techniques are a key operating mode for target compound quantitation with a triple quadrupole MS, providing sensitive and precise quantitative results by monitoring one or several primary ion transitions per targeted compound [85–87].

**Fig. 4:**
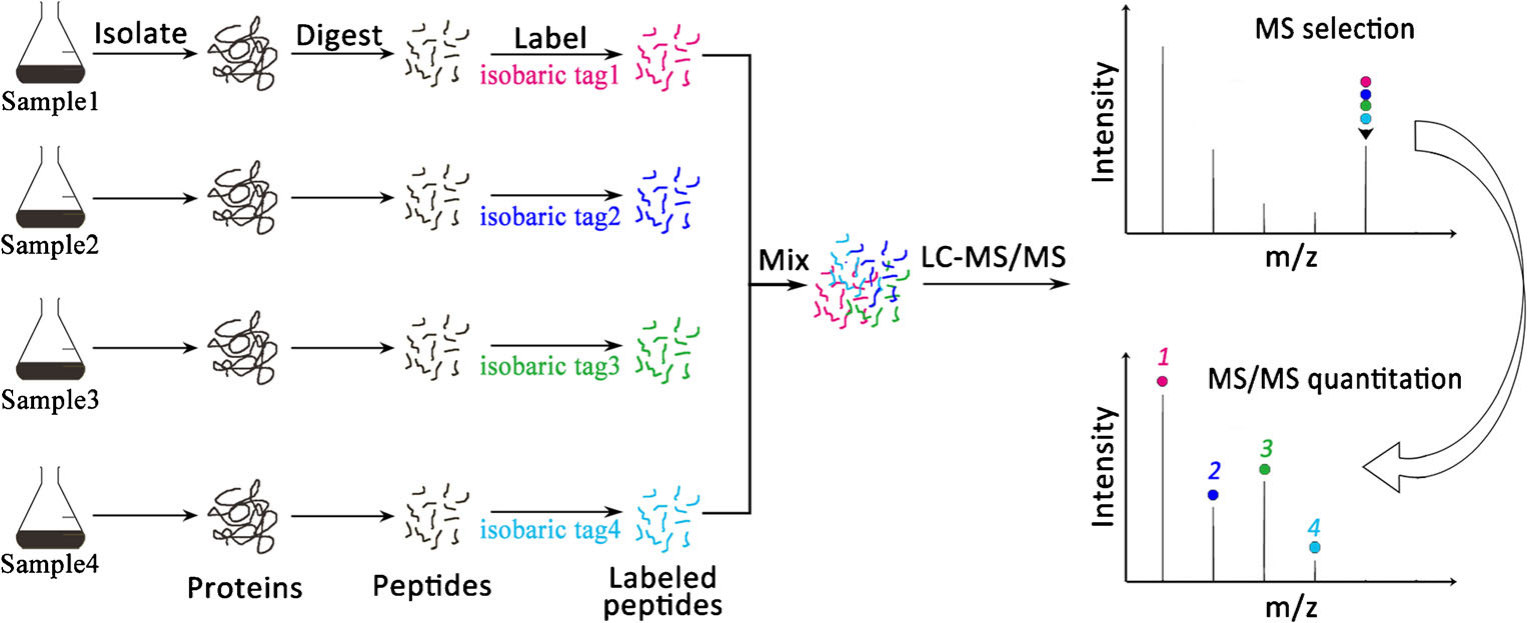
Isobaric chemical labeling method. Isobaric chemical labeling, including isobaric tags for relative and absolute quantification (iTRAQ) and tandem mass tag (TMT), labeled the N-termini and the lysine-side chains in the digested peptides with different isobaric compounds, which have the same mass and chemical structure but contain different numbers and combinations of ^13^C and ^15^N isotopes in the mass reporter. Then, the different tags were identified and the relative peptide abundances estimated. Because the masses of all of the tags are the same, identical peptides from different samples are co-eluted and selected by MS. After tag cleavage and another round of MS, the tags are used to quantitate relative peptide intensities, while the peptide fragment ions are sequenced for protein identification. The isobaric chemical labeling based multiplexing comparison is used to compare up to four, six, eight, or ten samples, depending the isobaric tags used (i.e., 4-plex iTRAQ, 6-plex TMT, 8-plex iTRAQ, or 10-plex TMT)

Besides, isobaric chemical labeling approaches employed multiplex isobaric mass tags and, thus, benefited from increased throughput potential (Fig 4). Isobaric tags for relative and absolute quantification (iTRAQ) [88] has become a popular method for quantitative proteomic labeling, in which trypsin-digested peptides were labeled separately with different isotopic variants of iTRAQ tags. Thus, the labeled peptides contained three functional parts: a reporter ion group, a mass normalization group, and an amine-reactive group [89]. By using an iTRAQ-based proteomic analysis, our group addressed the differential bacterial proteome of *S. aureus* to daptomycin antibiotic exposure [37], from which bacterial NDK and NT5 genes are indicators in response to the antibiotic treatment. This iTRAQ method could also be combined with SRM/MRM to perform a large-scale phosphoproteome analysis [90, 91].

The capability of multiplexing is its unique advantage of isobaric chemical labeling in comparison to the metabolic labeling techniques. Currently, iTRAQ 4-plex and 8-plex labeling reagents are commercially available, allowing to compare 2–8 samples in a single LC-MS/MS analysis. Similarly, another commercially available reagent termed tandem mass tag (TMT) is also ideal for multiplexed protein quantitation [92]. Moreover, TMT has currently been developed to a 10-plex set of tags. When analyzing more than eight samples, TMT 10-plex has the advantage of comparing up to ten samples simultaneously [93, 94]. One recent publication applied SPS-MS3 TMT10-plex analysis to investigate the proteomic alterations in *S. cerevisiae* resulting from the adaptation of yeast from glucose to nine different carbon sources [95], and over 5000 yeast proteins across ten growth conditions were quantified in a single experiment.

Comparing with the labeling methods, label-free quantification is simpler, more economical, and more applicable, without the requirement for extra preparation steps for labeling and the limitation for materials that cannot be directly labeled [96]. Label-free quantification tries to find the differences in protein abundances by integrating the aligned peak intensity profiles from LC-MS/MS analyses. One previous report compared the membrane proteomes between virulent *M. tuberculosis* H37Rv and the *Mycobacterium bovis* BCG vaccine strain by using label-free quantitative proteomics [97]. As a result, 2203 membrane-associated proteins were identified in high confidence and 294 of them showed statistically significant differences of at least two-fold in relative abundance, which is helpful to investigate mechanisms underlying *M. tuberculosis* H37Rv virulence and identify new targets for therapeutic intervention. After that, the role of the *M. tuberculosis* SecA2 pathway in exporting solute binding proteins and Mce transporters to the cell wall has been revealed recently [98].

However, some major bottlenecks still remain for this approach, such as the need for measuring samples under strict standard procedures, the restricted specific quantification not suitable as generic tools at a proteome scale, and the modest accuracy of the quantitative readouts not capable of the detection of small changes [99, 100]. Nevertheless, new algorithms like MaxLFQ [99] and aLFQ [101] were developed to solve those problems and to achieve the highest possible accuracy of quantification. Promisingly, sequential window acquisition of all theoretical mass spectra (SWATH) MS, a data-independent workflow that uses a first quadrupole isolation window to step across a mass range, collecting high-resolution full-scan composite MS/MS at each step and generating an ion map of fragments from all detectable precursor masses, is optimally suited to acquire proteome-wide quantitative data over many samples with a high degree of reproducibility, large dynamic range, and low limit of detection [102, 103]. A recent study applied SWATH MS to examine proteomic reorganization of *M. tuberculosis* during exponential growth, hypoxia-induced dormancy, and resuscitation. A dataset was obtained covering >2000 proteins revealing how protein biomass is distributed among cellular functions and the investigators provided a quantitative description of microbial states [104]. Alternatively, robust, highly parallel procedures to generate peptide mixtures are critical to increase effectiveness. For example, a method termed filter-aided sample preparation (FASP), combined with nano-LC in one dimension followed by online MS/MS analysis on a Q-exactive MS, can distinguish more than 1000 distinct microbial proteins and 1000 distinct human proteins from urine in a single experiment [105, 106].

## Perspective

With the development of proteomics and MS technology, more high-efficiency and high-throughput methods can be available for microorganism investigation in the future. Microbial proteomics provides a powerful tool for microbial basic research and translational applications, not only profiling the mechanism and microbial physiology research, but also giving a clue in clinical diagnosis and antimicrobial therapy. Moreover, it will be helpful for a better understanding of microbial community functions and microbial physiology, which provides tools to exploit novel bioactives and new antibiotics for clinical antimicrobial therapy.

## Abbreviations

FASP: Filter-aided sample preparation
iTRAQ: Isobaric tags for relative and absolute quantification
LC: Liquid chromatography
LC-MS/MS: Liquid chromatography tandem mass spectrometry
MALDI-TOF: Matrix-assisted laser desorption/ionization time-of-flight
MRM: Multiple reaction monitoring
MS: Mass spectrometry
SILAC: Stable isotope labeling with amino acids in cell culture
SRM: Selected reaction monitoring
SWATH: Sequential window acquisition of all theoretical mass spectra
2-DE: Two-dimensional gel electrophoresis
TMT: Tandem mass tag

## References

[1] Jones KE, Patel NG, Levy MA, Storeygard A, Balk D, Gittleman JL, Daszak P (2008). Global trends in emerging infectious diseases. Nature.

[2] Fournier P-E, Drancourt M, Colson P, Rolain J-M, La Scola B, Raoult D (2013). Modern clinical microbiology: new challenges and solutions. Nat Rev Microbiol.

[3] Schofield C (2015). Antibiotics: Current innovations and future trends. Edited by Sergio Sánchez and Arnold L. Demain. ChemMedChem.

[4] Arias CA, Murray BE (2009). Antibiotic-resistant bugs in the 21st century—a clinical super-challenge. N Engl J Med.

[5] Meyer B, Cookson B (2010). Does microbial resistance or adaptation to biocides create a hazard in infection prevention and control?. J Hosp Infect.

[6] Gould IM, Bal AM (2013). New antibiotic agents in the pipeline and how they can help overcome microbial resistance. Virulence.

[7] Walsh C (2003). Where will new antibiotics come from?. Nat Rev Microbiol.

[8] Vranakis I, Goniotakis I, Psaroulaki A, Sandalakis V, Tselentis Y, Gevaert K, Tsiotis G (2014). Proteome studies of bacterial antibiotic resistance mechanisms. J Proteomics.

[9] VerBerkmoes NC, Denef VJ, Hettich RL, Banfield JF (2009). Systems biology: functional analysis of natural microbial consortia using community proteomics. Nat Rev Microbiol.

[10] Tyers M, Mann M (2003). From genomics to proteomics. Nature.

[11] Otto A, Bernhardt J, Hecker M, Becher D (2012). Global relative and absolute quantitation in microbial proteomics. Curr Opin Microbiol.

[12] Zhang W, Li F, Nie L (2010). Integrating multiple ‘omics’ analysis for microbial biology: application and methodologies. Microbiology.

[13] Seneviratne CJ, Wang Y, Jin L, Wong SS, Herath TD, Samaranayake LP (2012). Unraveling the resistance of microbial biofilms: has proteomics been helpful?. Proteomics.

[14] Li W, Yao Z, Sun L, Hu W, Cao J, Lin W, Lin X (2016). Proteomics analysis reveals a potential antibiotic cocktail therapy strategy for *Aeromonas hydrophila* infection in biofilm. J Proteome Res.

[15] Soufi Y, Soufi B (2016). Mass spectrometry-based bacterial proteomics: focus on dermatologic microbial pathogens. Front Microbiol.

[16] Liang S, Shen G, Xu X, Xu Y, Wei Y (2009). Affinity purification combined with mass spectrometry-based proteomic strategy to study mammalian protein complex and protein–protein interactions. Curr Proteomics.

[17] Grassl N, Kulak NA, Pichler G, Geyer PE, Jung J, Schubert S, Sinitcyn P, Cox J, Mann M (2016). Ultra-deep and quantitative saliva proteome reveals dynamics of the oral microbiome. Genome Med.

[18] van der Poll T, Opal SM (2008). Host–pathogen interactions in sepsis. Lancet Infect Dis.

[19] Ferreira D, Seca AML, Diana CGA, Silva AMS (2016). Targeting human pathogenic bacteria by siderophores: a proteomics review. J Proteomics.

[20] Bartlett AH, Park PW (2010). Proteoglycans in host–pathogen interactions: molecular mechanisms and therapeutic implications. Expert Rev Mol Med.

[21] Lee EJ, Pontes MH, Groisman EA (2013). A bacterial virulence protein promotes pathogenicity by inhibiting the bacterium’s own F1Fo ATP synthase. Cell.

[22] Wei P, Wong WW, Park JS, Corcoran EE, Peisajovich SG, Onuffer JJ, Weiss A, Lim WA (2012). Bacterial virulence proteins as tools to rewire kinase pathways in yeast and immune cells. Nature.

[23] Otto A, van Dijl JM, Hecker M, Becher D (2014). The *Staphylococcus aureus* proteome. Int J Med Microbiol.

[24] Durmuş S, Çakır T, Özgür A, Guthke R (2015). A review on computational systems biology of pathogen–host interactions. Front Microbiol.

[25] Sun F, Ding Y, Ji Q, Liang Z, Deng X, Wong CC, Yi C, Zhang L, Xie S, Alvarez S, Hicks LM, Luo C, Jiang H, Lan L, He C (2012). Protein cysteine phosphorylation of SarA/MgrA family transcriptional regulators mediates bacterial virulence and antibiotic resistance. Proc Natl Acad Sci U S A.

[26] Meng Q, Liu P, Wang J, Wang Y, Hou L, Gu W, Wang W (2016). Systematic analysis of the lysine acetylome of the pathogenic bacterium *Spiroplasma eriocheiris* reveals acetylated proteins related to metabolism and helical structure. J Proteomics.

[27] Fu Y (2016). Data analysis strategies for protein modification identification. Methods Mol Biol.

[28] Calder B, Soares NC, de Kock E, Blackburn JM (2015). Mycobacterial proteomics: analysis of expressed proteomes and post-translational modifications to identify candidate virulence factors. Expert Rev Proteomics.

[29] Ren J, Sang Y, Tan Y, Tao J, Ni J, Liu S, Fan X, Zhao W, Lu J, Wu W, Yao YF (2016). Acetylation of lysine 201 inhibits the DNA-binding ability of PhoP to regulate *Salmonella* virulence. PLoS Pathog.

[30] Salgado P (2012) Host–pathogen interactions: insights at atomic level. ITQB Seminar. Available online at: http://www.itqb.unl.pt/events/seminars. Accessed 19 Dec 2012

[31] Spiller DG, Wood CD, Rand DA, White MR (2010). Measurement of single-cell dynamics. Nature.

[32] Martins BM, Locke JC (2015). Microbial individuality: how single-cell heterogeneity enables population level strategies. Curr Opin Microbiol.

[33] Ghaemmaghami S, Huh WK, Bower K, Howson RW, Belle A, Dephoure N, O’Shea EK, Weissman JS (2003) Global analysis of protein expression in yeast. Nature 425(6959):737–741 10.1038/nature02046 14562106

[34] Davies J (2011). How to discover new antibiotics: harvesting the parvome. Curr Opin Chem Biol.

[35] Ma W, Zhang D, Li G, Liu J, He G, Zhang P, Yang L, Zhu H, Xu N, Liang S (2016). Antibacterial mechanism of daptomycin antibiotic against *Staphylococcus aureus* based on a quantitative bacterial proteome analysis. J Proteomics.

[36] Pierce CG, Lopez-Ribot JL (2013). Candidiasis drug discovery and development: new approaches targeting virulence for discovering and identifying new drugs. Expert Opin Drug Discovery.

[37] Breker M, Gymrek M, Schuldiner M (2013). A novel single-cell screening platform reveals proteome plasticity during yeast stress responses. J Cell Biol.

[38] Yu Y, Sikorski P, Bowman-Gholston C, Cacciabeve N, Nelson KE, Pieper R (2015). Diagnosing inflammation and infection in the urinary system via proteomics. J Transl Med.

[39] Lima TB, Pinto MF, Ribeiro SM, de Lima LA, Viana JC, Gomes Júnior N, Cândido Ede S, Dias SC, Franco OL (2013). Bacterial resistance mechanism: what proteomics can elucidate. FASEB J.

[40] Yu Y, Pieper R (2015). Urinary pellet sample preparation for shotgun proteomic analysis of microbial infection and host–pathogen interactions. Methods Mol Biol.

[41] Suh MJ, Kuntumalla S, Yu Y, Pieper R (2014). Proteomes of pathogenic *Escherichia coli*/*Shigella* group surveyed in their host environments. Expert Rev Proteomics.

[42] Boots M, Hudson PJ, Sasaki A (2004). Large shifts in pathogen virulence relate to host population structure. Science.

[43] Peterson J, Garges S, Giovanni M, McInnes P, Wang L, Schloss JA, Bonazzi V, McEwen JE, Wetterstrand KA, Deal C, Baker CC, Di Francesco V, Howcroft TK, Karp RW, Lunsford RD, Wellington CR, Belachew T, Wright M, Giblin C, David H, Mills M, Salomon R, Mullins C, Akolkar B, Begg L, Davis C, Grandison L, Humble M, Khalsa J, Little AR, Peavy H, Pontzer C, Portnoy M, Sayre MH, Starke-Reed P, Zakhari S, Read J, Watson B, Guyer M, NIH HMP Working Group (2009). The NIH Human Microbiome Project. Genome Res.

[44] Wilmes P, Bond PL (2006). Metaproteomics: studying functional gene expression in microbial ecosystems. Trends Microbiol.

[45] Provenzano JC, Siqueira JF, Rôças IN, Domingues RR, Paes Leme AF, Silva MR (2013). Metaproteome analysis of endodontic infections in association with different clinical conditions. PLoS One.

[46] Schneider T, Riedel K (2010). Environmental proteomics: analysis of structure and function of microbial communities. Proteomics.

[47] Schneider T, Keiblinger KM, Schmid E, Sterflinger-Gleixner K, Ellersdorfer G, Roschitzki B, Richter A, Eberl L, Zechmeister-Boltenstern S, Riedel K (2012). Who is who in litter decomposition? metaproteomics reveals major microbial players and their biogeochemical functions. ISME J.

[48] Lassek C, Burghartz M, Chaves-Moreno D, Otto A, Hentschker C, Fuchs S, Bernhardt J, Jauregui R, Neubauer R, Becher D, Pieper DH, Jahn M, Jahn D, Riedel K (2015). A metaproteomics approach to elucidate host and pathogen protein expression during catheter-associated urinary tract infections (CAUTIs). Mol Cell Proteomics.

[49] Erickson AR, Cantarel BL, Lamendella R, Darzi Y, Mongodin EF, Pan C, Shah M, Halfvarson J, Tysk C, Henrissat B, Raes J, Verberkmoes NC, Fraser CM, Hettich RL, Jansson JK (2012). Integrated metagenomics/metaproteomics reveals human host–microbiota signatures of Crohn’s disease. PLoS One.

[50] Wang X, Zhang A, Sun H (2013). Power of metabolomics in diagnosis and biomarker discovery of hepatocellular carcinoma. Hepatology.

[51] Zhernakova A, Kurilshikov A, Bonder MJ, Tigchelaar EF, Schirmer M, Vatanen T, Mujagic Z, Vila AV, Falony G, Vieira-Silva S, Wang J, Imhann F, Brandsma E, Jankipersadsing SA, Joossens M, Cenit MC, Deelen P, Swertz MA, Weersma RK, Feskens EJ, Netea MG, Gevers D, Jonkers D, Franke L, Aulchenko YS, Huttenhower C, Raes J, Hofker MH, Xavier RJ, Wijmenga C, Fu J, LifeLines cohort study (2016). Population-based metagenomics analysis reveals markers for gut microbiome composition and diversity. Science.

[52] Falony G, Joossens M, Vieira-Silva S, Wang J, Darzi Y, Faust K, Kurilshikov A, Bonder MJ, Valles-Colomer M, Vandeputte D, Tito RY, Chaffron S, Rymenans L, Verspecht C, De Sutter L, Lima-Mendez G, D’hoe K, Jonckheere K, Homola D, Garcia R, Tigchelaar EF, Eeckhaudt L, Fu J, Henckaerts L, Zhernakova A, Wijmenga C, Raes J (2016). Population-level analysis of gut microbiome variation. Science.

[53] Baquero F, Nombela C (2012). The microbiome as a human organ. Clin Microbiol Infect.

[54] Clarke G, Stilling RM, Kennedy PJ, Stanton C, Cryan JF, Dinan TG (2014). Minireview: Gut microbiota: the neglected endocrine organ. Mol Endocrinol.

[55] Benakis C, Brea D, Caballero S, Faraco G, Moore J, Murphy M, Sita G, Racchumi G, Ling L, Pamer EG, Iadecola C, Anrather J (2016). Commensal microbiota affects ischemic stroke outcome by regulating intestinal gammadelta T cells. Nat Med.

[56] Nesvizhskii AI (2010). A survey of computational methods and error rate estimation procedures for peptide and protein identification in shotgun proteomics. J Proteomics.

[57] Nesvizhskii AI, Aebersold R (2005). Interpretation of shotgun proteomic data: the protein inference problem. Mol Cell Proteomics.

[58] Nesvizhskii AI (2014). Proteogenomics: concepts, applications and computational strategies. Nat Methods.

[59] Jaffe JD, Berg HC, Church GM (2004). Proteogenomic mapping as a complementary method to perform genome annotation. Proteomics.

[60] Ferro M, Tardif M, Reguer E, Cahuzac R, Bruley C, Vermat T, Nugues E, Vigouroux M, Vandenbrouck Y, Garin J, Viari A (2008) PepLine: a software pipeline for high-throughput direct mapping of tandem mass spectrometry data on genomic sequences. J Proteome Res 7(5):1873–1883 10.1021/pr070415k 18348511

[61] Pang CN, Tay AP, Aya C, Twine NA, Harkness L, Hart-Smith G, Chia SZ, Chen Z, Deshpande NP, Kaakoush NO, Mitchell HM, Kassem M, Wilkins MR (2014). Tools to covisualize and coanalyze proteomic data with genomes and transcriptomes: validation of genes and alternative mRNA splicing. J Proteome Res.

[62] Tovchigrechko A, Venepally P, Payne SH (2014). PGP: parallel prokaryotic proteogenomics pipeline for MPI clusters, high-throughput batch clusters and multicore workstations. Bioinformatics.

[63] Brouwer RW, van Hijum SA, Kuipers OP (2009). MINOMICS: visualizing prokaryote transcriptomics and proteomics data in a genomic context. Bioinformatics.

[64] Zhang J, Yang MK, Zeng H, Ge F (2016) GAPP: a proteogenomic software for genome annotation and global profiling of posttranslational modifications in prokaryotes. Mol Cell Proteomics pii: mcp.M116.060046 10.1074/mcp.M116.060046 PMC5098048 27630248

[65] Mutters NT, Hodiamont CJ, de Jong MD, Overmeijer HP, van den Boogaard M, Visser CE (2014). Performance of Kiestra total laboratory automation combined with MS in clinical microbiology practice. Ann Lab Med.

[66] Karlsson R, Gonzales-Siles L, Boulund F, Svensson-Stadler L, Skovbjerg S, Karlsson A, Davidson M, Hulth S, Kristiansson E, Moore ER (2015). Proteotyping: proteomic characterization, classification and identification of microorganisms—a prospectus. Syst Appl Microbiol.

[67] Sabbagh B, Mindt S, Neumaier M, Findeisen P (2016). Clinical applications of MS-based protein quantification. Proteomics Clin Appl.

[68] van Belkum A, Chatellier S, Girard V, Pincus D, Deol P, Dunne WM (2015). Progress in proteomics for clinical microbiology: MALDI-TOF MS for microbial species identification and more. Expert Rev Proteomics.

[69] Clark AE, Kaleta EJ, Arora A, Wolk DM (2013). Matrix-assisted laser desorption ionization-time of flight mass spectrometry: a fundamental shift in the routine practice of clinical microbiology. Clin Microbiol Rev.

[70] Singhal N, Kumar M, Kanaujia PK, Virdi JS (2015). MALDI-TOF mass spectrometry: an emerging technology for microbial identification and diagnosis. Front Microbiol.

[71] Urwyler SK, Glaubitz J (2016). Advantage of MALDI-TOF-MS over biochemical-based phenotyping for microbial identification illustrated on industrial applications. Lett Appl Microbiol.

[72] Spanu T, Posteraro B, Fiori B, D’Inzeo T, Campoli S, Ruggeri A, Tumbarello M, Canu G, Trecarichi EM, Parisi G, Tronci M, Sanguinetti M, Fadda G (2012). Direct MALDI-TOF mass spectrometry assay of blood culture broths for rapid identification of *Candida* species causing bloodstream infections: an observational study in two large microbiology laboratories. J Clin Microbiol.

[73] Kerns PW, Ackhart DF, Basaraba RJ, Leid JG, Shirtliff ME (2014). Mycobacterium tuberculosis pellicles express unique proteins recognized by the host humoral response. Pathog Dis.

[74] Guo H, Chen C, Lee DJ, Wang A, Ren N (2014). Proteomic analysis of sulfur–nitrogen–carbon removal by *Pseudomonas* sp. C27 under micro-aeration condition. Enzym Microb Technol.

[75] Xu C, Lin X, Ren H, Zhang Y, Wang S, Peng X (2006). Analysis of outer membrane proteome of *Escherichia coli* related to resistance to ampicillin and tetracycline. Proteomics.

[76] Riederer K, Cruz K, Shemes S, Szpunar S, Fishbain JT (2015). MALDI-TOF identification of Gram-negative bacteria directly from blood culture bottles containing charcoal: Sepsityper® kits versus centrifugation-filtration method. Diagn Microbiol Infect Dis.

[77] Zboromyrska Y, Rubio E, Alejo I, Vergara A, Mons A, Campo I, Bosch J, Marco F, Vila J (2016). Development of a new protocol for rapid bacterial identification and susceptibility testing directly from urine samples. Clin Microbiol Infect.

[78] Íñigo M, Coello A, Fernández-Rivas G, Rivaya B, Hidalgo J, Quesada MD, Ausina V (2016). Direct identification of urinary tract pathogens from urine samples, combining urine screening methods and matrix-assisted laser desorption ionization-time of flight mass spectrometry. J Clin Microbiol.

[79] Abhyankar W, de Koning LJ, Brul S, de Koster CG (2014). Spore proteomics: the past, present and the future. FEMS Microbiol Lett.

[80] Xian F, Zi J, Wang Q, Lou X, Sun H, Lin L, Hou G, Rao W, Yin C, Wu L, Li S, Liu S (2016). Peptide biosynthesis with stable isotope labeling from a cell-free expression system for targeted proteomics with absolute quantification. Mol Cell Proteomics.

[81] Hünten S, Kaller M, Drepper F, Oeljeklaus S, Bonfert T, Erhard F, Dueck A, Eichner N, Friedel CC, Meister G, Zimmer R, Warscheid B, Hermeking H (2015). p53-Regulated networks of protein, mRNA, miRNA, and lncRNA expression revealed by integrated pulsed stable isotope labeling with amino acids in cell culture (pSILAC) and next generation sequencing (NGS) analyses. Mol Cell Proteomics.

[82] Clark DJ, Fondrie WE, Yang A, Mao L (2016). Triple SILAC quantitative proteomic analysis reveals differential abundance of cell signaling proteins between normal and lung cancer-derived exosomes. J Proteomics.

[83] Li HY, Zhang LK, Zhu XJ, Shang J, Chen X, Zhu Y, Guo L (2015). Analysis of EV71 infection progression using triple-SILAC-based proteomics approach. Proteomics.

[84] Picotti P, Aebersold R (2012). Selected reaction monitoring-based proteomics: workflows, potential, pitfalls and future directions. Nat Methods.

[85] Keshishian H, Addona T, Burgess M, Kuhn E, Carr SA (2008). Quantitative, multiplexed assays for low abundance proteins in plasma by targeted mass spectrometry and stable isotope dilution. Mol Cell Proteomics.

[86] Zhi W, Wang M, She JX (2011). Selected reaction monitoring (SRM) mass spectrometry without isotope labeling can be used for rapid protein quantification. Rapid Commun Mass Spectrom.

[87] Zhou S, Hu Y, DeSantos-Garcia JL, Mechref Y (2015). Quantitation of permethylated N-Glycans through multiple-reaction monitoring (MRM) LC-MS/MS. J Am Soc Mass Spectrom.

[88] Zhou Y, Shan Y, Zhang L, Zhang Y (2014). Recent advances in stable isotope labeling based techniques for proteome relative quantification. J Chromatogr A.

[89] Li Z, Adams RM, Chourey K, Hurst GB, Hettich RL, Pan C (2012). Systematic comparison of label-free, metabolic labeling, and isobaric chemical labeling for quantitative proteomics on LTQ Orbitrap Velos. J Proteome Res.

[90] Adachi J, Narumi R, Tomonaga T (2016). Targeted phosphoproteome analysis using selected/multiple reaction monitoring (SRM/MRM). Methods Mol Biol.

[91] Narumi R, Tomonaga T (2016). Quantitative analysis of tissue samples by combining iTRAQ isobaric labeling with selected/multiple reaction monitoring (SRM/MRM). Methods Mol Biol.

[92] Thompson A, Schäfer J, Kuhn K, Kienle S, Schwarz J, Schmidt G, Neumann T, Johnstone R, Mohammed AK, Hamon C (2003) Tandem mass tags: a novel quantification strategy for comparative analysis of complex protein mixtures by MS/MS. Anal Chem 75(8):1895–1904 10.1021/ac0262560 12713048

[93] Skubitz APN, Afiuni S, Boylan KLM, Geller M, Argenta P, Hoffman S, Griffin T (2016). Abstract B34: Tandem Mass Tag 10-plex isobaric labeling of Pap test proteins: a novel method for the identification of ovarian cancer protein biomarkers by mass spectrometry. Clin Cancer Res.

[94] Zhang Z, Yang X, Mirokhin YA, Tchekhovskoi DV, Ji W, Markey SP, Roth J, Neta P, Hizal DB, Bowen MA, Stein SE (2016). Interconversion of peptide mass spectral libraries derivatized with iTRAQ or TMT labels. J Proteome Res.

[95] Paulo JA, O’Connell JD, Everley RA, O’Brien J, Gygi MA, Gygi SP (2016). Quantitative mass spectrometry-based multiplexing compares the abundance of 5000 *S. cerevisiae* proteins across 10 carbon sources. J Proteomics.

[96] Lee HC, Chen CY, Au LC (2011). Systemic comparison of repression activity for miRNA and siRNA associated with different types of target sequences. Biochem Biophys Res Commun.

[97] Gunawardena HP, Feltcher ME, Wrobel JA, Gu S, Braunstein M, Chen X (2013). Comparison of the membrane proteome of virulent *Mycobacterium tuberculosis* and the attenuated *Mycobacterium bovis* BCG vaccine strain by label-free quantitative proteomics. J Proteome Res.

[98] Feltcher ME, Gunawardena HP, Zulauf KE, Malik S, Griffin JE, Sassetti CM, Chen X, Braunstein M (2015). Label-free quantitative proteomics reveals a role for the *Mycobacterium tuberculosis* SecA2 pathway in exporting solute binding proteins and Mce transporters to the cell wall. Mol Cell Proteomics.

[99] Cox J, Hein MY, Luber CA, Paron I, Nagaraj N, Mann M (2014). Accurate proteome-wide label-free quantification by delayed normalization and maximal peptide ratio extraction, termed MaxLFQ. Mol Cell Proteomics.

[100] Schmidt C, Grønborg M, Deckert J, Bessonov S, Conrad T, Lührmann R, Urlaub H (2014). Mass spectrometry-based relative quantification of proteins in precatalytic and catalytically active spliceosomes by metabolic labeling (SILAC), chemical labeling (iTRAQ), and label-free spectral count. RNA.

[101] Rosenberger G, Ludwig C, Röst HL, Aebersold R, Malmström L (2014). aLFQ: an R-package for estimating absolute protein quantities from label-free LC-MS/MS proteomics data. Bioinformatics.

[102] Sidoli S, Lin S, Xiong L, Bhanu NV, Karch KR, Johansen E, Hunter C, Mollah S, Garcia BA (2015). Sequential Window Acquisition of all Theoretical Mass Spectra (SWATH) analysis for characterization and quantification of histone post-translational modifications. Mol Cell Proteomics.

[103] Shang S, Monfregola M, Caruthers M (2016) Peptide-substituted oligonucleotide synthesis and non-toxic, passive cell delivery. Signal Transduction and Targeted Therapy 1:16019 10.1038/sigtrans.2016.19 PMC5661639 29263901

[104] Schubert Olga T, Ludwig C, Kogadeeva M, Zimmermann M, Rosenberger G, Gengenbacher M, Gillet Ludovic C, Collins Ben C, Röst Hannes L, Kaufmann Stefan HE, Sauer U, Aebersold R (2015). Absolute proteome composition and dynamics during dormancy and resuscitation of *Mycobacterium tuberculosis*. Cell Host Microbe.

[105] Yu Y, Pieper R (2015). Urine sample preparation in 96-well filter plates to characterize inflammatory and infectious diseases of the urinary tract. Adv Exp Med Biol.

[106] Yu Y, Suh MJ, Sikorski P, Kwon K, Nelson KE, Pieper R (2014). Urine sample preparation in 96-well filter plates for quantitative clinical proteomics. Anal Chem.

